# Testing a novel isokinetic dynamometer constructed using a 1080 Quantum

**DOI:** 10.1371/journal.pone.0201179

**Published:** 2018-07-20

**Authors:** Alanna K. Whinton, Kyle M. A. Thompson, Geoffrey A. Power, Jamie F. Burr

**Affiliations:** 1 Department of Human Health and Nutritional Sciences, Human Performance and Health Research Laboratory, University of Guelph, Guelph, Ontario, Canada; 2 Department of Human Health and Nutritional Sciences, Neuromechanical Performance Research Laboratory, University of Guelph, Guelph, Ontario, Canada; Univerzitet u Beogradu, SERBIA

## Abstract

This study sought to assess the reliability and comparability of two custom-built isokinetic dynamometers (Model A and Model B) with the gold-standard (Humac Norm). The two custom-built dynamometers consisted of commercially available leg extension machines attached to a robotically controlled resistance device (1080 Quantum), able to measure power, force and velocity outputs. Twenty subjects (14m/6f, 26±4.8yr, 176±7cm, 74.4±12.4kg) performed concentric leg extensions on the custom-built dynamometers and the Humac Norm. Fifteen maximal leg extensions were performed with each leg at 180° s^-1^, or the linear equivalent (~0.5m s^-1^). Peak power (W), mean power (W), and fatigue indexes (%) achieved on all three devices were compared. Both custom-built dynamometers revealed high reliability for peak and mean power on repeated tests (ICC>0.88). Coefficient of variation (CV) and standard error of measurement (SEM) were small when comparing power outputs obtained using Model A and the Humac Norm ($\bar{x}$ CV = 9.0%, $\bar{x}$ SEM = 49W; peak CV = 8.4%, peak SEM = 49W). Whereas, Model B had greater variance ($\bar{x}$ CV = 13.3% $\bar{x}$ SEM = 120W; peak CV = 14.7%, peak SEM = 146W). The custom-built dynamometers are capable of highly reliable measures, but absolute power outputs varied depending on the leg extension model. Consistent use of a single model offers reliable results for tracking muscular performance over time or testing an intervention.

## Introduction

The quantification of muscular strength and endurance is important in clinical testing [1,2], athletic capacity assessment [3,4], and broadly within human research in the exercise sciences [5,6]. Reliable and valid measures are required for the assessment of standardized test values with normative data, to track changes over time, and to interpret these effects with reference to a significant and meaningful change.

Using isokinetic dynamometry, the power a muscle group generates can be quantified throughout its full range of motion by employing an accommodating resistance to a standardized contraction velocity [5,7,8]. As such, isokinetic dynamometry provides a highly reproducible measure of neuromuscular performance in both health and disease [9]. Parameters such as peak force, mean force, power, and angular work can be derived through relatively straight-forward maximal or submaximal protocols [5,6,10]. Tight controls requiring precise movement and standard operating procedures allow for tracking of differences due to subject variation, rather than inconsistent data capture [11].

Recently, a novel linear resistance machine, called the 1080 Quantum (1080 Motion, Lidingö, Sweden), has been developed for applications in the sport-training and rehabilitation fields. The 1080 Quantum employs a robotically controlled dynamic cable resistance that permits the targeting of resistive forces to emphasize loading or unloading at different movement phases across fixed or dynamic velocities (concentric and eccentric). This is accomplished by information of voltage and current being relayed to the servo motor to calculate the torque delivered to the motor shaft [12]. A high resolution (20 bit) optical encoder is attached to the motor, measuring the exact position of the motor axis, providing precise speed values while the line is being unwound from the drum [12]. The 1080 Quantum has previously shown valid and reproducible results when measuring force, power and speed [12–14]. While the intended application of the 1080 Quantum is targeted toward dynamic multi-joint, or rotational movements, the cable resistance offers the possibility of the attachment to a single-joint resistance exercise machine, allowing the testing of power about a single joint or muscle group. As such, when connected to the appropriate piece of “selectorized” equipment in place of the normal iso-inertial resistance of the machine (weight stack), the 1080 Quantum may be capable of measurements similar to those collected using established isokinetic dynamometers, though this has not been previously tested. The application of such a versatile training and testing device could offer many benefits, amongst them being the ability to construct a task-specific dynamometer for a far lower cost.

The primary focus of the current study is to demonstrate that a custom-built isokinetic dynamometer, which is similarly reliable as the gold-standard Humac Norm and could be employed for human physiology research, could be created using commercially available exercise equipment. Our secondary aims include, establishing the relationship in power outcomes and indexes of muscle fatigue between the custom-built dynamometers and the Humac Norm, as well as, determining whether measurement outcomes such as peak and mean power on the 1080 Quantum remain reliable when connected to different leg extension machines. It was hypothesized that irrespective of the exercise equipment used to control the exercise motion, test-retest data would demonstrate a highly reliable measurement using the 1080 Quantum as an isokinetic resistance. Secondly, it was hypothesized that measures using the custom-built isokinetic dynamometers would have a standardized degree of offset to those collected on a Humac Norm dynamometer, which could be adjusted for using a device specific mathematical correction factor. To aid in the comparisons of the different dynamometers, regression equations for the specific equipment tests were generated, but that there would be some degree of offset between the measurements as the Humac Norm measures torque, while the custom-built dynamometers measures linear force. Lastly, it was hypothesized that employing two different leg extension machines connected to the 1080 Quantum would alter the degree of offset with the Humac Norm, owing to design differences such as adjustability of lever arms from the point of rotation, and the shape of the cam around which the cable runs between the point of attachment and resistance.

## Methods

### Subjects

Twenty healthy, recreationally active males (n = 14) and females (n = 6) (26 ± 4.8 years, 175.7 ± 7.4 cm in height, 74.4 ± 12.4 kg of body mass) participated in the study, with sample size based on previous reliability literature using leg extensions [5,10,15]. Exclusion criteria were limited to the presence of a significant medical disorder that would compromise the subject’s safety (e.g. chronic disease, musculoskeletal or cardiovascular complications). Subjects were instructed to maintain their regular eating habits and physical activity, while avoiding intense physical activity during the 2 days prior to testing. All subjects provided written informed consent prior to study participation, and the rights of the subjects were protected throughout the study in accordance with the ethical guidelines of the University of Guelph Research Ethics Board, who approved the protocol (REB#16MY024).

### Instrumentation

Three isokinetic dynamometers, the two, custom-built isokinetic dynamometers (Model A and Model B) and the Humac Norm (CSMi Solutions, Stoughton, MA), were used for the assessment of isokinetic leg power and fatigue index for both legs in the study. The 1080 Quantum was attached, in turn, to two different leg extension machines, Model A (the Element Fitness Carbon Dual 9019 Leg Extension/Leg Curl; The Treadmill Factory Mississauga, Canada), or Model B (IT9328 Leg Extension/Leg Curl; Viva Fitness, United States), which were similar in function but differed in their design. The newly constructed isokinetic dynamometers have similar features and outcome measures to the Humac Norm, when used for leg extension exercises; and thus, allows the custom-built isokinetic dynamometers to be compared to the Humac Norm.

### Set-up

**Fig 1:**
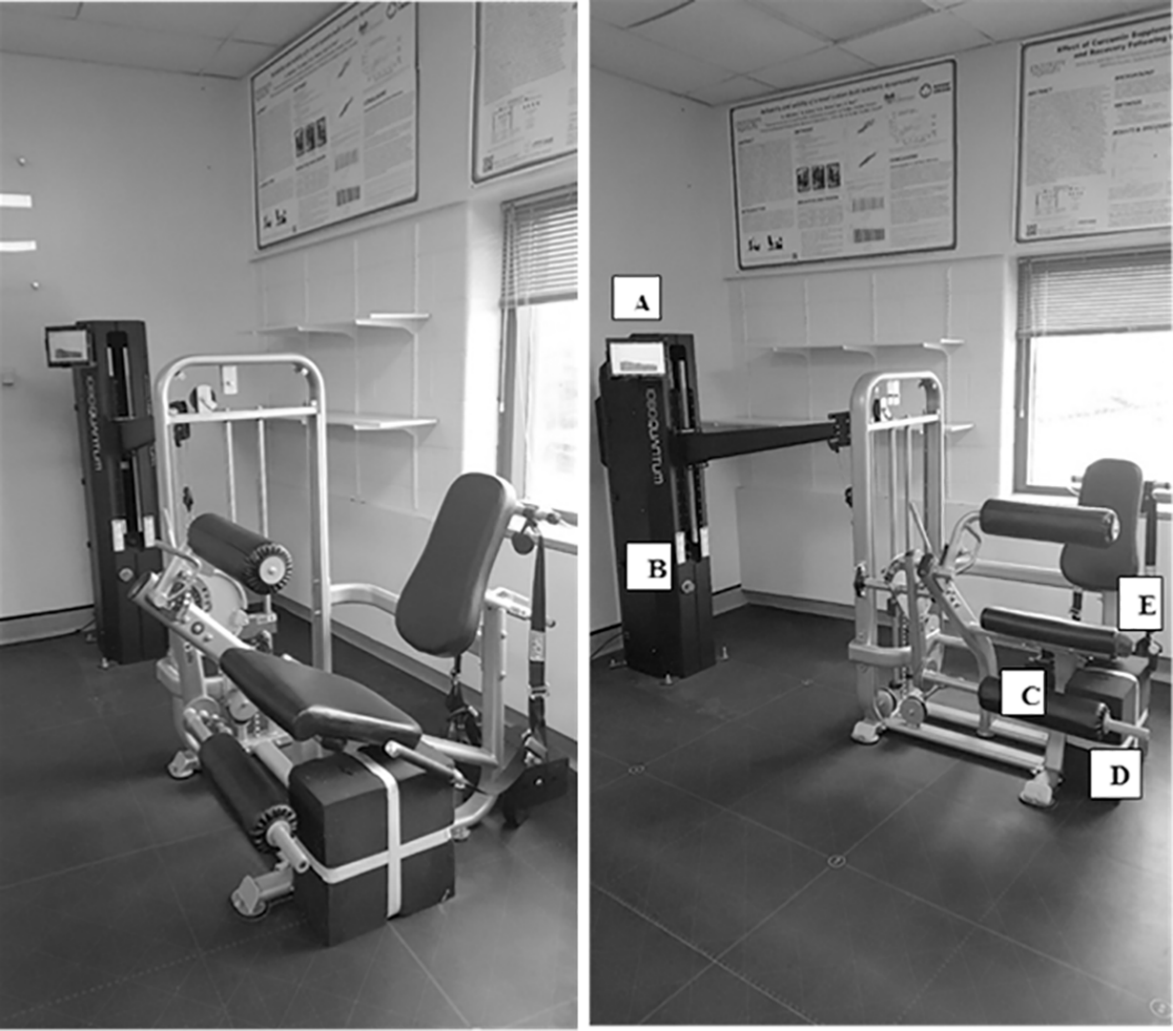
Configuration of the 1080 Quantum attached to Model A leg extension. The power outputs (W) would be presented on the **A.** tablet, calculated from the **B.** 1080 Quantum. The participant would sit in the leg extension machine and kick the **C.** movement arm outwards to complete the leg extension. The **D.** range of motion apparatus was in place to suspend the extension, bringing the participant’s leg back to the neutral position to be prepared for subsequent extensions. Finally, the participant was secured with a **E.** harness.

**Fig 2:**
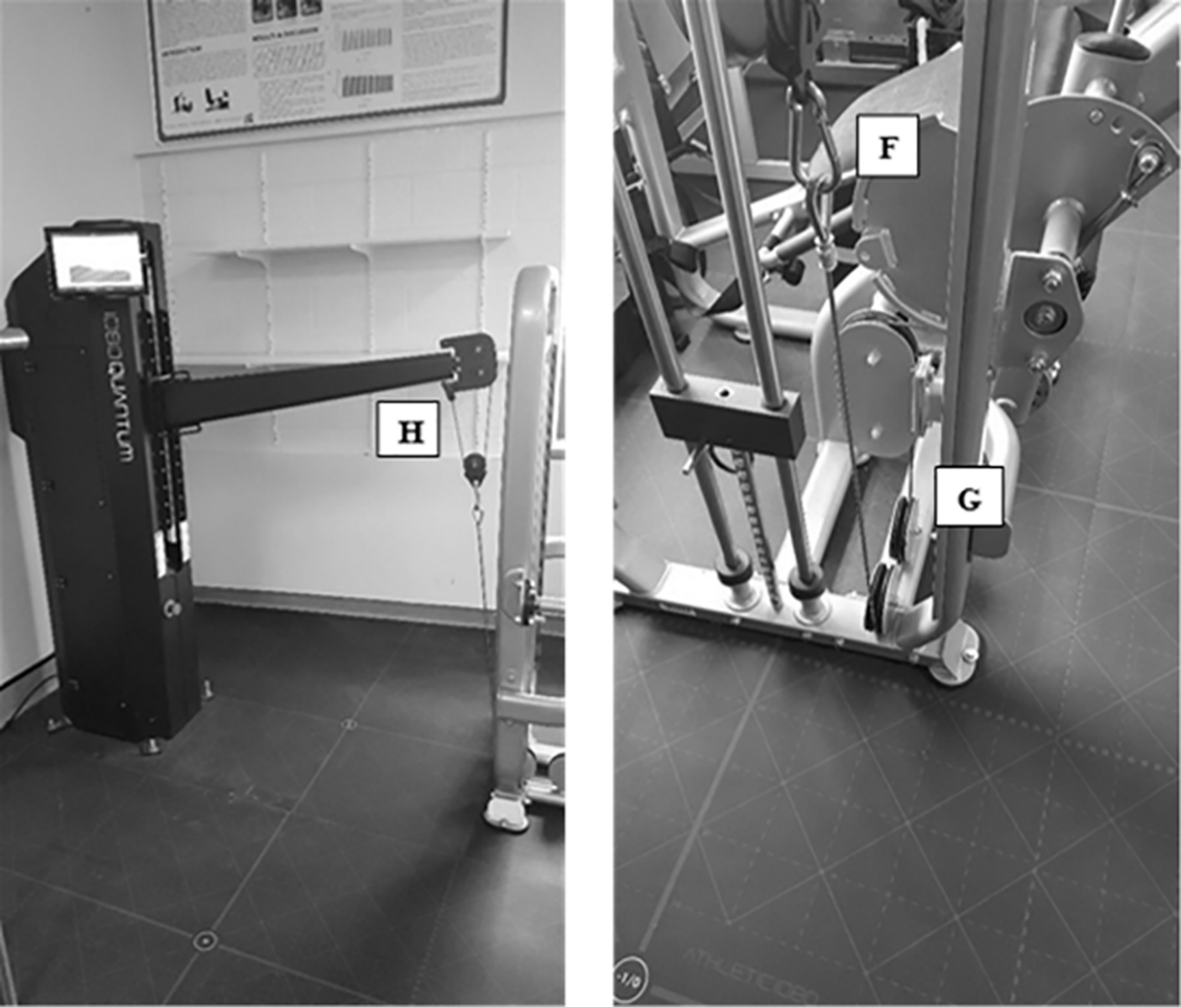
Continuation of configuration of the 1080 Quantum attached to Model A leg extension. The leg extension machine was attached to the **H.** 1080 Quantum by a **F.** carabiner through a **G.** custom-fit cable.

To create the custom-built dynamometers, the 1080 Quantum was connected to a commercial leg extension machine by removing the original weight stack. More specifically, a custom fit cable was attached to the cam of the leg extension, through the incorporated pulley and connected to a carabiner at the end of the line for the 1080 Quantum, so that the 1080 Quantum was able to manipulate the actions of the leg extension (Figs 1 and 2). Subsequently, calibration was completed daily per the manufacturer’s instructions. The 1080 Quantum was operated with 5 kg (for female subjects) and 8 kg (for male subjects) added to the concentric and eccentric load. Incorporation of these loads was crucial to the operation of the 1080 Quantum, to keep the cables taught and in the pulleys and for an initial load against which to push. On all devices, subjects were seated comfortably, restrained using a chest harness and lap cushion to limit any movement other than the leg extension task, and a distal shin pad was placed 2 cm above the lateral malleolus of the tested leg. The knee joint was aligned with the axis of rotation to the mechanical dynamometer. A goniometer was used to verify the starting position of 90° flexion at the knee, with a stop put in place to control range of motion. Once the subject was positioned correctly, all adjustable variables were recorded for standardization between trials. The maximal achievable linear velocity of the 1080 Quantum dynamometers were set to match the 180° s^-1^ angular velocity used during the exercise trial conducted on the Humac Norm. The following equation was used to match the linear to angular velocity; υ = *r*ω, where υ represents velocity in metres per second, *r* represents the length from the femoral epicondyle to the distal shin pad (radius in metres) and ω represents angular velocity in radians per second.

### Exercise protocol

**Fig 3:**
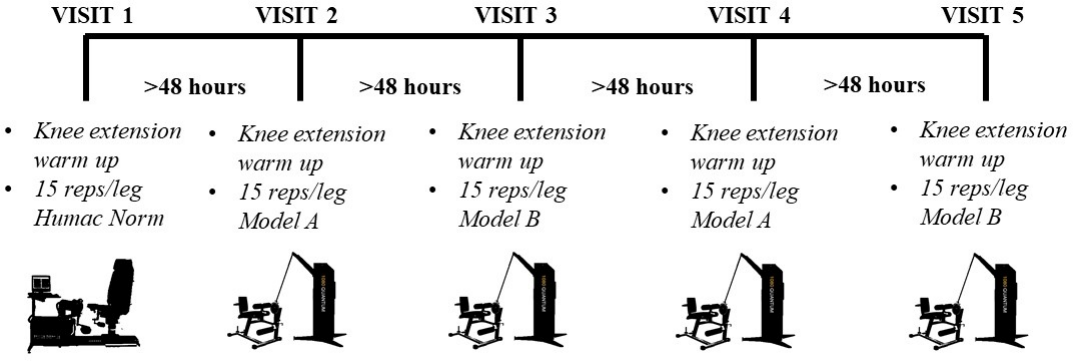
Schematic timeline of experimental protocol. Warm-up of both legs was initiated before the exercise protocol of 15 maximal concentric leg extensions at an equivalent of 180° s^-1^ on both legs at each visit. Repeated tests were performed on Model A and Model B, with a single test performed on the Humac Norm, separated by at least 48 hours.

Prior to data collection, participants warmed up both legs with light leg extensions until they were prepared to perform maximally. Subjects were then allowed to perform maximal leg extensions until a marked increase in power output between repetitions was no longer observed. This warm-up and familiarization protocol was performed prior to all five testing sessions (1x Humac Norm, 2 x Model A, 2 x Model B) (for experimental protocol schematic, refer to Fig 3) to reduce the potential learning effect of performing maximal leg extensions on the different dynamometers [16]. Three to five minutes rest was then allotted to each subject prior to the exercise protocol to ensure they were not fatigued. Subjects performed 15 maximal effort, 180° s^-1^ equivalent leg extensions (from 90° knee flexion to 0°) per leg, with a 1s passive return (manually assisted by tester) to the starting position. A 10-minute rest period was given following completion of 15 repetitions on the first leg before testing the second leg. The 10-minute rest period was chosen to avoid any fatigue related cross-over artifact, and for sufficient time to adjust the Humac Norm for testing the opposite leg (set-up according to Dalton et al. 2015) [17]. The order in which a subject’s legs were tested was randomized preceding the visit and kept constant for all subsequent visits. Throughout all trials strong verbal encouragement and real-time visual feedback of leg extension power outputs was provided to encourage maximal power production. A single test was performed on the Humac Norm, while test-retest was performed using Model A and B of the 1080 Quantum using a repeated test separated by at least 48 hours and performed at the same time of day.

Measures were recorded and analyzed with LabChart (Labchart, Pro Modules 2014, version 8) software for the Humac Norm and integrated 1080 Motion software for the Quantum. Torque, position and angular velocity data were sampled at 2500 Hz and digitized by a 16-bit analog-to-digital system (PowerLab Data Acquisition Unit 16/35, AD Instruments, Bela Vista, New South Wales, Australia) on the Humac Norm. Force, power, speed and work were sampled at 111 Hz and computed on the 1080 Motion (Version 3, Lidingö, Sweden). Power was calculated as the product of torque multiplied by angular velocity on the Humac Norm. The values obtained were taken at the highest point of the single contraction and was recorded as peak power, for each device. Mean power was calculated by taking the sum of the contractions and dividing it by the number of contractions performed (i.e. 15), per leg. Fatigue index was determined across individual contractions as: $fatigue\;index=(\frac{\bar{\chi }\;first\;5\;contractions‑\bar{\chi }\;last\;5\;contractions}{\bar{\chi }\;first\;5\;contractions})x\;100$. All supplementary material is provided in S1 Table.

### Statistical analysis

For assessment of reliability; peak power, mean power and fatigue index were compared between the two repeated tests of the two, custom-built isokinetic dynamometers using a 2 (variation of the 1080 Quantum: Model A and Model B) x 2 (test: first test and second test) ANOVA with Bonferroni *post-hoc* tests. Pearson correlations were used to compare Model A and Model B. Reliability of measures for repeated tests on the custom-built isokinetic dynamometers were further examined using intra-class correlation coefficient (ICC_2,1_) and was classified according to the categories of Sole and colleagues (2007) [10]. All procedures were reproduced for the 1080 Quantum using a second leg extension attachment, and additional comparisons were drawn between 1080 Quantum Model A and Model B using the same statistical tests. As raw values only indicate precision, comparability of the custom isokinetic dynamometers vs the Humac Norm was further assessed by examining the coefficient of variation (CV) and through standard error of measurement (SEM) (with 95% confidence intervals), indicating the standard deviation of scores between the two tests of the Humac Norm and the associated model. CV values below 1.15 are considered acceptable based on Stokes (1985) [18] and Santos and colleagues (2013) [19]. All statistical procedures were performed with SPSS 24 statistical software (SPSS Inc., Chicago IL, USA), or publicly available spreadsheets (sportssci.org) for verification of reliability and comparability and an *alpha* of p<0.05 was set *a priori*.

## Results

### Reliability

Repeated tests on the custom-built isokinetic dynamometers did not differ in measures of peak power, mean power or fatigue index (Table 1). These findings were consistent for Model A and Model B. Also, irrespective of either custom-built isokinetic dynamometer (Model A or Model B), ICC for both peak and mean power were high. Significant differences were found between Model A and Model B for both peak power (Δ225W ± 88W) and mean power (Δ202W ± 79W), with higher raw values consistently generated on Model B (*p*<0.0001).

**Table 1:** Reliability of measures between two tests (pre and post) of 15 leg extensions per leg on two variations of a custom-built isokinetic dynamometer, the 1080 Quantum.

	Mean ± SD (W)	*p*	ICC	SEM (W)	Correlation
**Peak Power (W)**					
Model A_1_	344 ± 110	*0*.*1*^**†**^	0.88 (0.78–0.93)	38.3 (32.5–47.1)	98^*^
Model A_2_	339 ± 110				
*Model B*_*1*_	569 ± 187^**§**^	*0*.*96*^**‡**^	0.91 (0.82–0.95)	55.7 (47.4–68.7)	97^*^
*Model B*_*2*_	569 ± 177				
**Mean Power (W)**					
Model A_1_	296 ± 96	*0*.*06*^**†**^	0.88 (0.78–0.93)	34.1 (28.8–41.7)	98^*^
Model A_2_	286 ± 99				
*Model B*_*1*_	498 ± 163^**§**^	*0*.*6*^**‡**^	0.91 (0.83–2395)	47.8 (40.7–59)	98^*^
*Model B*_*2*_	501 ± 155				
**Fatigue Index (%)**					
Model A_1_	17.8 ± 6.2%	*0*.*14*^**†**^	0.09 (-0.36–0.5)	5.50% (4.4–7.4%)	17
Model A_2_	20.3 ± 5.2%				
*Model B*_*1*_	14.2 ± 3.8%^**§**^	*0*.*7*^**‡**^	0.51 (0.08–0.76)	2.80% (2.2–3.8%)	48^*^
*Model B*_*2*_	14.6 ± 4.3%				

**A**_**1**_ **=** test 1 on leg extension attachment (Model A) of the Quantum; **A**_**2**_ **=** test 2 on leg extension attachment (Model A) of the Quantum; **B**_**1**_ **=** test 1 on leg extension attachment (Model B) of the Quantum; **B**_**2**_ **=** test 2 on leg extension attachment (Model B) of the Quantum; **SD =** standard deviation; **ICC =** intra-class correlation coefficient; **SEM =** standard error of measurement with 95% confidence intervals; ***p* =** between tests within each model

^*^ = <0.05

^**†**^ = comparison between Model A_1_ to Model A_2_

^**‡**^ = comparison between Model B_1_ to Model B_2_

^**§**^ = <0.05, difference between Model A_1_ to Model B_1_

### Comparability

**Fig 4:**
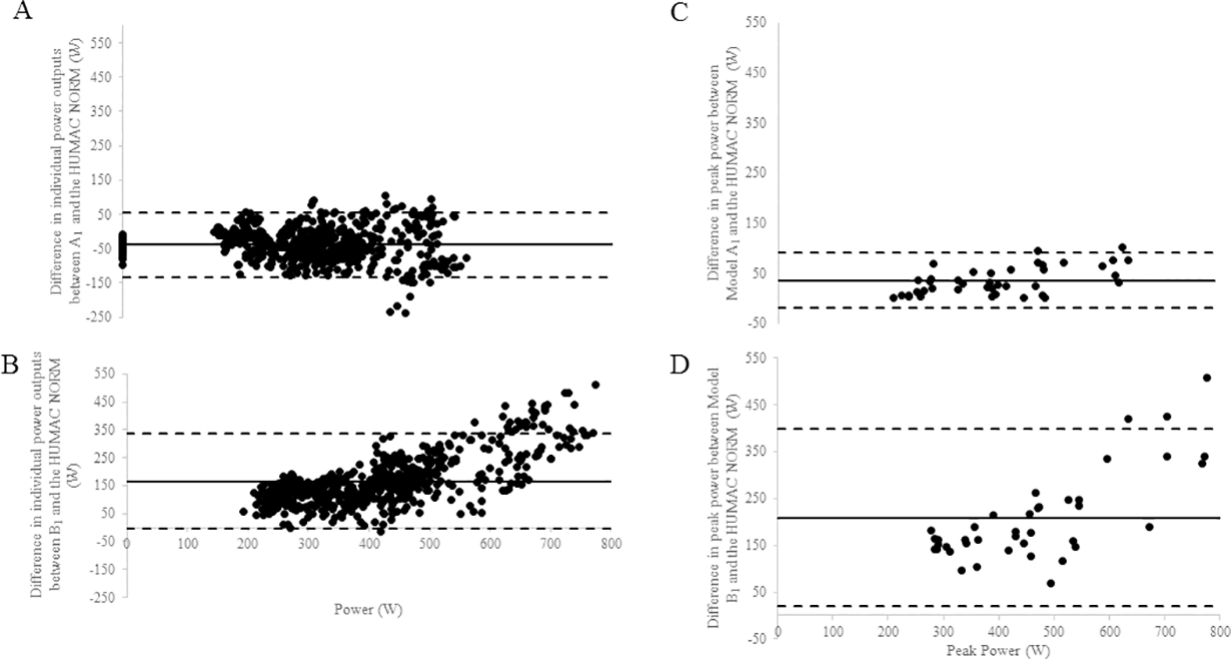
**Bland-Altman plots of difference in individual power output (W) between A) Model A**_**1**_ **and the Humac Norm and B) Model B**_**1**_ **and the Humac Norm.** Differences in peak power (W) between C) Model A_1_ and the Humac Norm and D) Model B_1_ and the Humac Norm. The horizontal lines represent the mean bias (solid black line) and upper and lower 95% limits of agreement (dotted black lines). The *y* axis is the difference of scores between machines and the *x* axis display the mean differences of those scores.

Comparison of both models (A and B) of the 1080 Quantum to the Humac Norm were significantly different for all assessments of peak power, mean power and fatigue index (*p* < 0.0001; Table 2). Log-transformed Bland-Altman plots are presented in Fig 4, displaying the agreement between Model A_1_ and the Humac Norm, and Model B_1_ and Humac Norm.

**Table 2:** Comparison of measures using the first test of 15 leg extensions per leg between a gold standard dynamometer (Humac Norm) and two variations of a custom-built isokinetic dynamometer (1080 Quantum).

	Mean ± SD (W)	*p*	CV (%)	SEM (W)	Correlation (R)
**Peak Power (W)**					
Humac Norm	361± 116				
Model A_1_	344 ± 110	0.015^||^	8.4	48.5	93^*^
Model B_1_	570 ± 187	<0.0001^**¶**^	14.7	146.1	90^*^
**Mean Power (W)**					
Humac Norm	333 ± 107				
Model A_1_	296 ± 96	<0.0001^||^	9.0	49.4	93^*^
Model B_1_	498 ± 163	<0.0001^**¶**^	13.3	119.6	92^*^
**Fatigue Index (%)**					
Humac Norm	5.7 ± 4.7%				
Model A_1_	17.8 ± 6.2%	<0.0001^||^	41.86	7.7%	21
Model B_1_	14.2 ± 3.8%	<0.0001^**¶**^	46.01	6.0%	-16

**A**_**1**_ **=** test 1 on Model A of the Quantum; **B**_**1**_ **=** test 1 on Model B of the Quantum; **SD =** standard deviation; **CV =** coefficient of variation; **SEM =** standard error of measurement; ***p* =** Comparison of each model of the Quantum to the Humac Norm

^*^ = <0.05

^**||**^ = comparison between the Humac Norm and Model A_1_

^**¶**^ = comparison between the Humac Norm and Model B_1_

## Discussion

The major finding of the present study was pairing of single-joint leg extension machines with the 1080 Quantum (Model A and Model B) showed strong consistency of test-retest (A_1_- A_2_; B_1_-B_2_) power outputs. This consistency was further indicated through a high peak and mean power ICC of 0.88 and 0.91 for Models A and B, respectively. In comparison, the gold standard dynamometers, the Humac Norm and the Cybex II have an ICC of ~0.84 [20] and 0.87 [21] for concentric leg extensions performed at the velocity used in the current study. These results demonstrate that the 1080 Quantum can be used in combination with readily available exercise equipment as a reliable and cost-effective dynamometer when completing test-retest measurements.

The comparability of power outputs between the Humac Norm and 1080 Quantum (Model A, test A_1_) revealed values of variation below 1.15 (<15%) and minimal error (SEM = ~49 W) (Table 2) [10]. The calculated indices of comparability, CV% and SEM, further substantiate the intra-machine reproducibility for peak and mean power of both tests. When comparing high quality, commercially available dynamometers, a variance of 6.25%-9.5% is typically observed [5,19,22], with low SEM values demonstrating more precise power output values when comparing between machines [23]. While it was not a primary purpose of the current paper to compare validity measures, it is interesting to note that the outputs obtained using Model A and the Humac Norm are within the variance observed in previous work [5,19,22] (A-Humac Norm: peak power = 8.4%, $\bar{x}$ power = 9.0%) (Table 2). SEM revealed the same peak (49 W) and mean power (49 W) values between Model A and the Humac Norm. This same amount of error indicates a strong parallel alignment and clear consistency of the Model A apparatus and the Humac Norm, even with different absolute values computed. The Bland-Altman plots (Fig 4) illustrate the variability and systematic bias between the power outputs of Model A and the Humac Norm. It is apparent that the agreement between Model A and the Humac Norm is strong, as the differences in individual power outputs are clustered around the mean and close to zero.

Comparison of peak power, mean power and fatigue index between Model A and the Humac Norm, displayed clear differences in absolute values. These findings were expected as the Humac Norm and the custom-built isokinetic dynamometers (Model A and Model B) are relatively distinct in respect to mechanical structure, adjustability, requirement for a baseline load and interfacing software, which influence force producing lever arm capabilities and data sampling rates (Humac Norm: 2500 Hz; 1080 Quantum: 111 Hz). To expand, the way in which the dynamometers are loaded for resistance is slightly different. While both devices were configured for isokinetic measurements, the custom-built dynamometer requires a small baseline load to be present even though the load is varied to ensure velocity is held constant with varied force produced by the participant. The requirement for a small degree of loading is to ensure that tension is kept in the cable pulleys, and to allow the unit to return to the starting position (which would be accomplished using gravity for a traditional isotonic machine). This load is not cumbersome, but would minimally alter the sensation of each extension, potentially leading to the observed changes in fatigue index. In addition, the 1080 Quantum does not have a controlled range of motion like the Humac Norm and was, thus, validated by a hand-held goniometer. Furthermore, the addition of a crescent shaped cam (incorporated in the leg extension) provides a potential mechanical advantage by distributing varied resistance throughout the range of motion to all for maximum force production of the muscle [9,24]. Different cam structures can result in dissimilar peak and mean power between machines depending on where the cam applies resistance and assistance [24] and, thus, may also alter fatigue index.

To verify the differences of power outputs as a result of different design and cam shapes, a second leg extension machine (Model B), manufactured with an oval-shaped cam, was attached to the 1080 Quantum, following the same design and statistical analysis as used with Model A. Findings of reliability between pre and post-tests (B_1_ –B_2_) of Model B of the 1080 Quantum, mimicked Model A’s consistency and ICC. Both peak and mean power output from the first test of Model B (B_1_) were within an acceptable range of variation, according to Stokes (1985) [18], when compared to the Humac Norm (CV%—peak = 14.7%; $\bar{x}$ = 13.3%), albeit at the higher end. In addition, the error of measurement between Model B_1_ and the Humac Norm was larger compared to Model A_1_ and the Humac Norm. Of importance, higher raw values for peak and mean power were produced in Model B_1_ compared to Model A_1_. This observation can be attributed to the different cam design, which allowed the muscles to be stressed in a different manner; making the exercise easier to perform at the weakest joint angles and applying maximized force wherein the muscle is the strongest, for equal relative loading [25].

Despite small differences between the custom-built and commercially available dynamometer, it is apparent that the 1080 Quantum combined with an existing exercise machine allows reliable determination of power production. Similarly, Papadopoulos and Stasinopoulos [26] reported comparable results (ICC = 0.98) when examining leg extensions on their dynamometer, however, it produced significantly different outputs than the Humac Norm. It stands to reasons that as long as these custom-built dynamometers are reliable within the machine, they should be able to be used in a research or rehabilitative setting. However, when comparing values between different types of dynamometers, or even the same dynamometer with different settings, it stands to reason that a mathematical adjustment specific to the mechanics of each machine could be introduced to provide comparison across instruments (Model A: *y* = 0.9752*x*+45.536; Model B: *y* = 0.5688*x*+49.252). This would, of course, only be necessary for comparisons of data collected using different devices or settings. Future directions include, assessment of the reliability and comparability of the 1080 Quantum with other fitness equipment, such as upper body exercise, to understand the applicability for testing different movements.

The 1080 Quantum dynamometer demonstrated reliable peak and mean power measures of concentric leg extensions at a commonly employed testing speed. While raw outputs differed from the gold-standard, the differences were strongly correlated and consistent for within-machine comparisons, suggesting the variation was introduced by the design of the leg extension machine. This was confirmed by our follow-up, showing a differently designed leg extension model altered this relationship. The test-retest reliability when using a single device was high, indicating potential for use in a variety of applications such as, monitoring return-to-play, rehabilitation and research.

## Supporting information

S1 Table SSPS study outcome measures. Raw data of peak power, mean power and muscle fatigue outputs for both models (A and B) of the Quantum and Humac Norm.
(SAV)
